# Case report: Navigating treatment pathways for cardiac intimal sarcoma with *PDGFRβ* N666K mutation

**DOI:** 10.3389/fonc.2024.1362347

**Published:** 2024-04-05

**Authors:** Akihiro Nishiyama, Shigeki Sato, Hiroyuki Sakaguchi, Hiroshi Kotani, Kaname Yamashita, Koushiro Ohtsubo, Keishi Mizuguchi, Hiroko Ikeda, Kenji Iino, Hirofumi Takemura, Shinji Takeuchi

**Affiliations:** ^1^ Department of Medical Oncology, Kanazawa University Hospital, Kanazawa, Japan; ^2^ Department of Diagnostic Pathology, Kanazawa University Hospital, Kanazawa, Japan; ^3^ Department of Cardiovascular Surgery, Kanazawa University, Kanazawa, Japan

**Keywords:** intimal sarcoma, *PDGFRβ* N666K mutation, precision oncology, MDM2 amplification, CDK4 amplification

## Abstract

In the realm of rare cardiac tumors, intimal sarcoma presents a formidable challenge, often requiring innovative treatment approaches. This case report presents a unique instance of primary intimal sarcoma in the left atrium, underscoring the critical role of genomic profiling in guiding treatment. Initial genomic testing unveiled a somatic, active mutation in *PDGFRβ* (*PDGFRβ* N666K), accompanied by *MDM2* and *CDK4* amplifications. This discovery directed the treatment course toward pazopanib, a PDGFRβ inhibitor, following irradiation. The patient’s response was remarkable, with the therapeutic efficacy of pazopanib lasting for 16.3 months. However, the patient experienced a recurrence in the left atrium, where subsequent genomic analysis revealed the absence of the *PDGFRβ* N666K mutation and a significant reduction in PDGFRβ expression. This case report illustrates the complexities and evolving nature of cardiac intimal sarcoma treatment, emphasizing the potential of PDGFRβ signaling as a strategic target and highlighting the importance of adapting treatment pathways in response to genetic shifts.

## 1 Introduction

Primary cardiac tumors are rare, with an incidence of 0.001%–0.003% worldwide (1), and intimal sarcoma is the most frequent subtype of cardiac sarcoma (2). Intimal sarcoma occurs in the innermost layer of large vessels, such as the pulmonary arteries. The clinical outcome is poor, with a median survival of only 12–13 months after radical surgery (3); thus, the development of effective treatment for intimal sarcoma is an unmet need. Gene profiling analyses of intimal sarcoma revealed that *MDM2* amplification and/or overexpression is frequently detected and is now considered one of the criteria for diagnosing intimal sarcoma (2). Moreover, a previous case report suggested that PDGFRβ may be involved in the tumorigenesis of intimal sarcoma (4). However, organized data on genetic findings of cardiac intimal sarcoma are limited.

## 2 Case description

**Figure 1:**
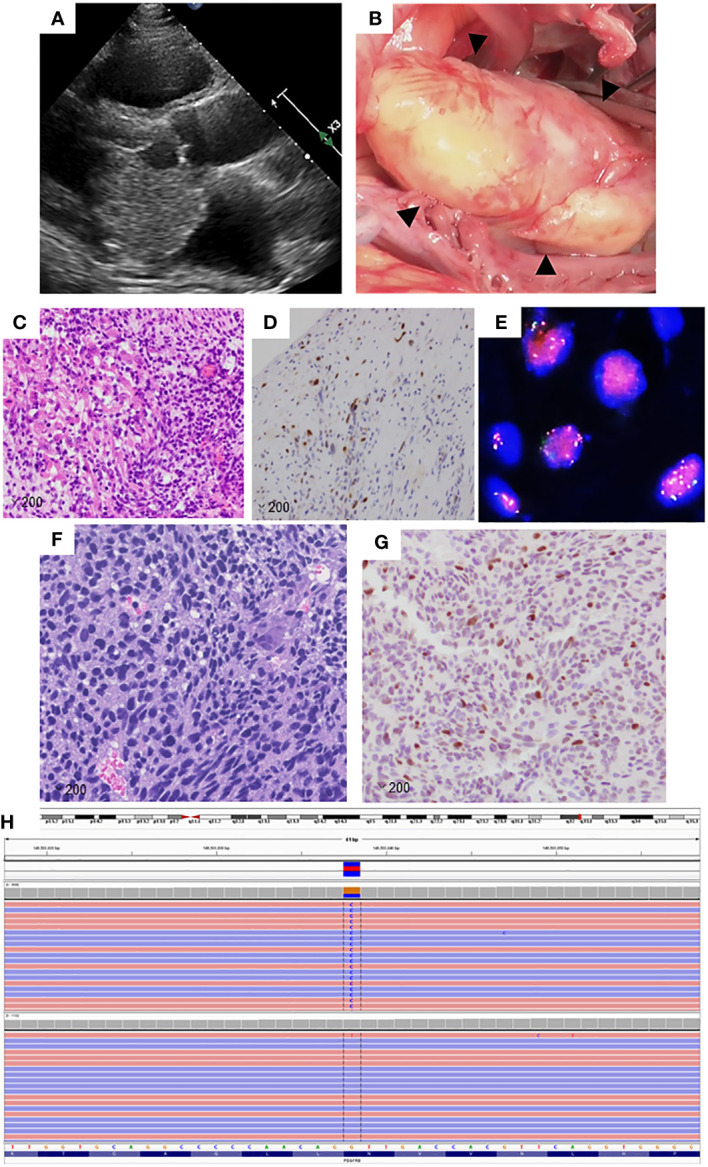
**(A)** Transthoracic echocardiography image of the primary lesion in the left atrium. **(B)** Perioperative image of the primary lesion in the left atrium. **(C)** The primary lesion shows the proliferation of spindle cells with a high nuclear-cytoplasmic ratio, magnification: ×200. **(D)** Tumor cells in the primary lesion showed positive immunoreactivity for MDM2 (magnification: ×200). **(E)** Fluorescence *in situ* hybridization analysis revealed the amplification of MDM2. Signals for MDM2 are presented in red, while those of CEP 12 are shown in green. **(F)** The left tailor muscle metastatic lesion consists of spindle cells and resembles the primary lesion (magnification: ×200). **(G)** The tumor cells in the metastatic lesion showed positive immunoreactivity for MDM2, magnification: ×200. **(H)** Illustration of *PDGFRβ* ^N666K^ using a next-generation sequencing platform as visualized in the Integrative Genomics Viewer.

A 45-year-old male patient, experiencing constitutional symptoms including palpitations and cough for four months, was admitted to Kanazawa University Hospital. Transthoracic echocardiography revealed a 5-cm-sized mass in the left atrium, possibly adhering to the mitral valve leaflets (**Figure 1A**). This mass resulted in pulmonary hypertension and low cardiac output. Emergent surgical excision of the mass was performed (**Figure 1B**), and pathological examination confirmed the diagnosis of intimal sarcoma in the left atrium, characterized by *MDM2* amplification and overexpression (**Figures 1C–E**).

Subsequently, the patient developed brain metastasis and underwent two sessions of Gamma Knife therapy. Despite these treatments, multiple bone metastases emerged, necessitating chemotherapy with doxorubicin, followed by eribulin. Although these regimens effectively controlled the bone metastases, a new lesion appeared in the left tailor muscle. A biopsy of this muscle mass was performed for comprehensive genomic testing. Histological analysis confirmed the left tailor muscle tumor as a metastatic intimal sarcoma, also displaying MDM2 overexpression (**Figures 1F, G**). The patient underwent palliative radiation therapy (60 Gy) targeting the left tailor muscle metastasis and continued with eribulin therapy. However, the thoracolumbar and sternal metastases progressed, manifesting as new lesions.

**Figure 2:**
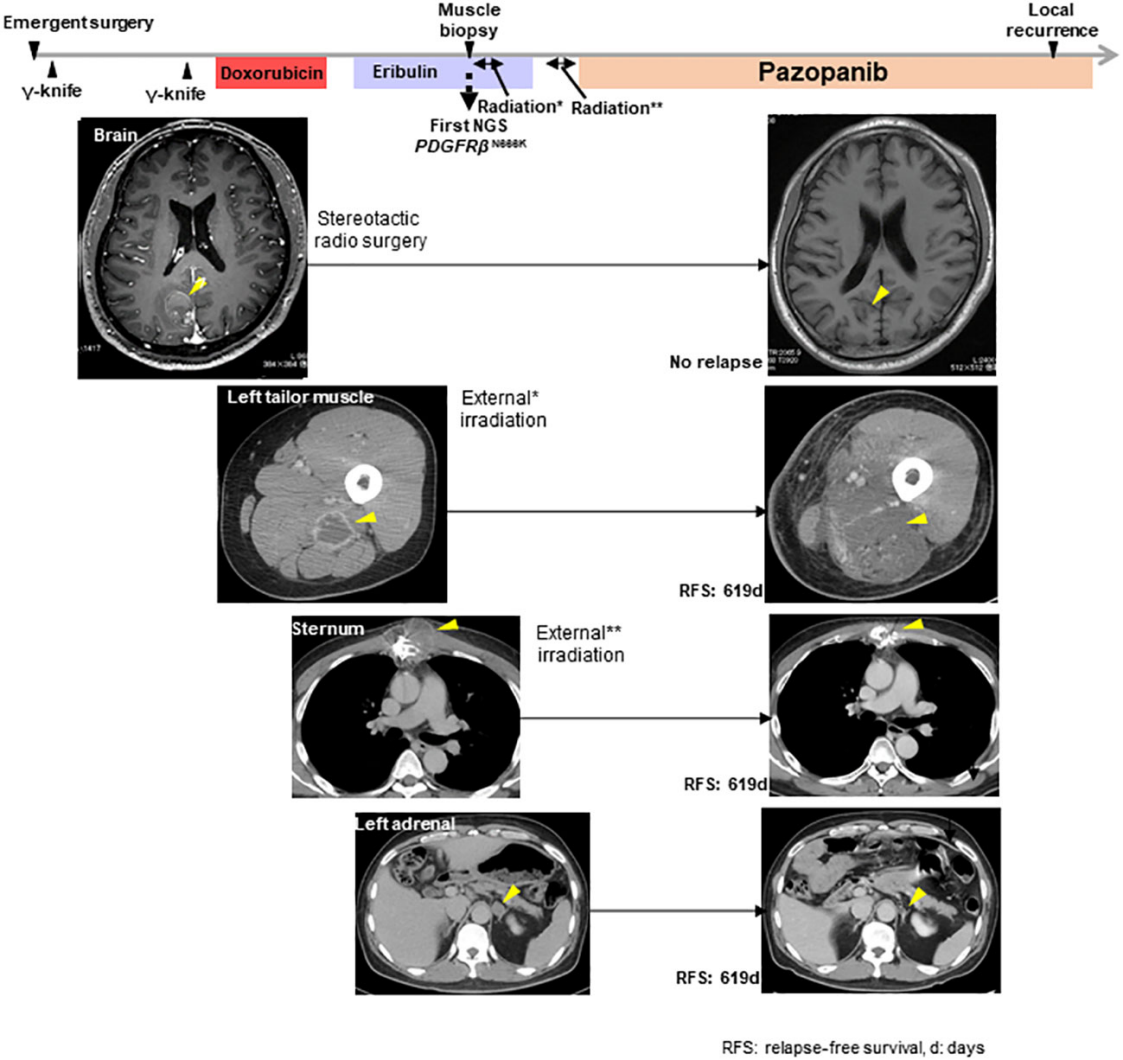
The clinical course from the initial operation to the local recurrence in the left atrium and the response of metastatic lesions to radiation or pazopanib. *indicated the external irradiation delivered to the left tailor muscle lesion. **indicated the external irradiation delivered to the sternum lesion. The yellow arrowheads show the target lesions.

Genomic testing of the left tail muscle metastasis revealed the presence of *PDGFRβ* N666K mutation, along with the amplification of *MDM2* and *CDK4*, both located on the long arm of chromosome 12 (**Figure 1H**). The *PDGFRβ* N666K had an allele frequency of 35.9%, and the copy numbers for *MDM2* and *CDK4* were 8.48 and 9.09, respectively. Due to pain resulting from the thoracolumbar and sternal metastases, the patient underwent external irradiation (30 Gy). Pazopanib, a sarcoma-approved drug active against PDGFRs, was initiated as a third-line treatment. Prior to the induction of pazopanib, the patient had several irradiated lesions, including metastases in the brain, left tailor muscle, sternum, and thoracolumbar spine, and a non-irradiated left adrenal metastasis. Pazopanib demonstrated a favorable and prolonged effect without requiring dose reduction, with the only side effect being a change in hair color. Unfortunately, 16.3 months after pazopanib initiation, a local recurrence in the left atrium was observed. For non-brain metastatic lesions, the relapse-free survival period was 619 days (**Figure 2**).

Salvage surgery was performed to remove the recurrent lesion, but local recurrence was detected after one and a half months. Owing to the inability to undergo another surgery, the patient received palliative radiation therapy for the treatment of a recurrent lesion in the left atrium (**Supplementary Figure 1A**). Further genomic testing on the left atrium recurrence sample (**Table 1**) resulted in the establishment of a patient-derived cell line from this sample, after obtaining the ethical review approval and patient consent (**Supplementary Figure 1B**). The recurrent lesion lacked the *PDGFRβ* N666K mutation and showed minimal PDGFRβ expression, in contrast to the clear PDGFRβ positivity observed in the primary and left tail muscle metastasis lesions (**Supplementary Figures 2A-C**).

**Table 1:** Difference between first genomic test results and second genomic test results by next-generation sequencing.

Detected Gene alterations	VAF or Copy number	
	1^st^ NGS	2^nd^ NGS
***PDGFRβ* N666K**	35.9%	Not detected
***MDM2* amplification**	CN 8.48	CN 33.5
***CDK4* amplification**	CN 9.09	CN 7.07
***CDKN2A* loss**	Not detected	Detected
***TP53* A198V**	47.5%	50.1%

NGS, next-generation sequencing; VAF, variant allele frequency; CN, copy number.

Subsequently, new metastatic lesions developed in the right atrium and the greater curvature of the stomach. The patient received palliative radiotherapy to prevent sudden death and gastrointestinal hemorrhage. Ultimately, disease progression occurred 44.6 months after the initial surgery.

## 3 Discussion

This case of cardiac intimal sarcoma with the *PDGFRβ* N666K mutation provides significant insights into the molecular landscape of this rare tumor type. Neuville et al.’s study on 42 cardiac intimal sarcomas reported MDM2 overexpression and *MDM2*/*CDK4* amplification (2). Additionally, *PDGFRβ* mutations, found in 15.3% of patients with intimal sarcomas, have been linked to oncogenesis in mesenchymal tumors (4–6). Notably, *PDGFRβ* D850V, M772V, R709H, and E472D mutations in intimal sarcoma have been previously documented (4, 5, 7). However, the *PDGFRβ* N666K mutation detected in our patient is the first instance in intimal sarcoma, underscoring its novelty and importance.

The oncogenic role of the PDGFRβ signaling pathway is further supported by the response of *PDGFRβ* N666K-transfected Ba/F3 cells to inhibitors like imatinib, nilotinib, or ponatinib, and its ability to induce cancer *in vivo* (6). The presence of *PDGFRβ* N666K in the primary lesion and the left tail muscle metastasis, as evidenced by the clear PDGFRβ positivity on the cell membrane (**Supplementary Figures 2A, B**), suggests that these lesions were driven by this mutation. However, the recurrence’s resistance to pazopanib and the lack of *PDGFRβ* N666K mutation therein indicate a complex and evolving tumor biology.

**Table 2** compares the efficacy of pazopanib in the present patient with those used in previous case reports (8–11). Results showed a significantly longer progression-free survival and better response, suggesting that *PDGFRβ* mutations may be a viable therapeutic target. However, the *CDK4* amplification and loss of CDKN2A observed in the recurrent lesion, coupled with increased sensitivity to abemaciclib, a CDK4/6 inhibitor, over pazopanib (**Supplementary Figure 1C**), highlight the necessity for tailored treatment approaches (12, 13). Although MDM2 acts as a negative regulator of TP53 by promoting its degradation and inhibiting tumor suppressor activity, the presence of TP53 mutations complicates the therapeutic landscape. Even if MDM2 inhibitors successfully block the MDM2-TP53 interaction, they are incapable of restoring TP53 function due to these underlying *TP53* alterations. Regrettably, the presence of a TP53 pathogenic variant precluded participation in studies involving MDM2 inhibitors such as milademetan (14), further emphasizing the need for a focus on alternative pathways influencing the cell cycle, specifically through *CDK4* amplification and *CDKN2A* loss, as pivotal resistance mechanisms.

**Table 2:** Efficacy of pazopanib as treatment for intimal sarcoma in the present and previous case reports.

Authors	Age (years)/sex	Gene alteration	Line of pazopanib	Response rate	Progression-free survival
Funatsu et al. (8)	71/F	Not reported	2nd	PR	2.0 months
Kollar et al. (9)	67.2^*^ M/F 1/1	Not reported	2nd	PR	Not reported
Sai et al. (10)	33/F	Not reported	1st	SD	5.8 months
Frezza et al. (11)	51^*^ M/F 7/5	Not reported	1st/2nd/further 1/3/8	PR/SD/PD 1/4/7	3.7^*^ months
Present case	46/M	*PDGFRβ* ^N666K^	3rd	CR	16.3 months

*Median number.

CR, complete response; PR, partial response; SD, stable disease; PD, progressive disease.

Our study has some limitations. Pazopanib is a multi-kinase inhibitor, which could obscure the specific impact of the drug on the *PDGFRβ* N666K mutation (10). Furthermore, as four lesions, excluding the left adrenal metastasis, had undergone irradiation before pazopanib administration, the distinct effect of the drug on these lesions remains uncertain.

## 4 Conclusion

This case highlights the potential of precision medicine for cardiac intimal sarcoma, showcasing the benefits of genomic testing in identifying specific alterations such as *PDGFRβ* N666K, *MDM2* amplification, and *CDK4* amplification. The prolonged efficacy observed with pazopanib following irradiation in a *PDGFRβ* N666K-positive patient emphasizes the potential of targeted therapies in improving the prognosis of patients with cardiac intimal sarcoma. This case report underscores the evolving nature of cancer treatment and the necessity for individualized therapeutic strategies.

## Data availability statement

The original contributions presented in the study are included in the article/**Supplementary Material**, further inquiries can be directed to the corresponding author/s.

## Ethics statement

Written informed consent was obtained from the individual(s) for the publication of any potentially identifiable images or data included in this article.

## Author contributions

AN: Writing – original draft, Writing – review & editing. SS: Writing – review & editing. HS: Writing – review & editing. HK: Writing – review & editing. KY: Writing – review & editing. KO: Writing – review & editing. KM: Writing – review & editing. HI: Writing – review & editing. KI: Writing – review & editing. HT: Writing – review & editing. ST: Writing – review & editing.

## Supplementary material

Supplementary Figure 1 The radiographic findings of the local recurrence in the left atrium and the experiment using the recurrence tissue. **(A)** The yellow arrowheads indicated the recurrence lesions. These lesions responded to palliative radiotherapy. **(B)** The appearance of the cell line established from the recurrence tissue. **(C)** The sensitivity of the patient-derived cell line to pazopanib or abemaciclib was determined through cell viability assays.

Supplementary Figure 2 Immunohistochemistry staining of *PDGFRβ* (Cell Signaling Technology; PDGF Receptor β (C82A3) Rabbit mAb #4564). **(A)** The primary lesion exhibited an adequate expression of *PDGFRβ*. **(B)** The left tailor muscle metastasis lesion demonstrated a high expression of *PDGFRβ*. **(C)** The local recurrence lesion in the left atrium had a low expression of *PDGFRβ*.

## References

[1] Neragi-Miandoab S Kim J Vlahakes GJ. Malignant tumours of the heart: a review of tumour type, diagnosis and therapy. Clin Oncol. (2007) 19:748–56. doi: 10.1016/j.clon.2007.06.009 17693068

[2] Neuville A Collin F Bruneval P Parrens M Thivolet F Gomez-Brouchet A. Intimal sarcoma is the most frequent primary cardiac sarcoma: clinicopathologic and molecular retrospective analysis of 100 primary cardiac sarcomas. Am J Surg Pathol. (2014) 38:461–9. doi: 10.1097/PAS.0000000000000184 24625414

[3] Dewaele B Floris G Finalet-Ferreiro J Fletcher CD Coindre JM Guillou L. Coactivated platelet-derived growth factor receptor α and epidermal growth factor receptor are potential therapeutic targets in intimal sarcoma. Cancer Res. (2010) 70:7304–14. doi: 10.1158/0008-5472.CAN-10-1543 20685895

[4] Roszik J Khan A Conley AP Livingston JA Groisberg R Ravi V. Unique aberrations in intimal sarcoma identified by next-generation sequencing as potential therapy targets. Cancers (Basel). (2019) 11:1283. doi: 10.3390/cancers11091283 31480474 PMC6770224

[5] Ito Y Maeda D Yoshida M Yoshida A Kudo-Asabe Y Nanjyo H. Cardiac intimal sarcoma with *PDGFRβ* mutation and co-amplification of *PDGFRα* and *MDM2*: an autopsy case analyzed by whole-exome sequencing. Virchows Arch. (2017) 471:423–8. doi: 10.1007/s00428-017-2135-x 28474091

[6] Arts FA Chand D Pecquet C Velghe AI Constantinescu S Hallberg B. PDGFRB mutants found in patients with familial infantile myofibromatosis or overgrowth syndrome are oncogenic and sensitive to imatinib. Oncogene. (2016) 35:3239–48. doi: 10.1038/onc.2015.383 26455322

[7] Fu X Niu W Li J Kiliti AJ Al-Ahmadie HA Iyer G. Activating mutation of PDGFRB gene in a rare cardiac undifferentiated intimal sarcoma of the left atrium: a case report. Oncotarget. (2017) 8:81709–16. doi: 10.18632/oncotarget.20700 PMC5655321 29113426

[8] Funatsu Y Hirayama M Shiraishi J Asakura T Wakaki M Yamada E. Intimal sarcoma of the pulmonary artery treated with pazopanib. Intern Med. (2016) 55:2197–202. doi: 10.2169/internalmedicine.55.6199 27522994

[9] Kollár A Jones RL Stacchiotti S Gelderblom H Guida M Grignani G. Pazopanib in advanced vascular sarcomas: an EORTC Soft Tissue and Bone Sarcoma Group (STBSG) retrospective analysis. Acta Oncol. (2017) 56:88–92. doi: 10.1080/0284186X.2016.1234068 27838944

[10] Sai S Imamura Y Kiyota N Jimbo N Toyoda M Funakoshi Y. Relationship between PDGFR expression and the response to pazopanib in intimal sarcoma of the pulmonary artery: a case report. Mol Clin Oncol. (2021) 14:6. doi: 10.3892/mco.2020.2168 33262886 PMC7690247

[11] Frezza AM Assi T Lo Vullo SL Ben-Ami E Dufresne A Yonemori K. Systemic treatments in MDM2 positive intimal sarcoma: a multicentre experience with anthracycline, gemcitabine, and pazopanib within the World Sarcoma Network. Cancer. (2020) 126:98–104. doi: 10.1002/cncr.32508 31536651 PMC9187112

[12] Dickson MA Schwartz GK Keohan ML D’Angelo SP Gounder MM Chi P. Phase 2 trial of the CDK4 inhibitor palbociclib (PD0332991) at 125 mg dose in well-differentiated or dedifferentiated liposarcoma. JAMA Oncol. (2016) 2:937–40. doi: 10.1001/jamaoncol.2016.0264 PMC4991028 27124835

[13] Fennell DA King A Mohammed S Greystoke A Anthony S Poile C. Abemaciclib in patients with p16ink4A-deficient mesothelioma (MiST2): a single-arm, open-label, phase 2 trial. Lancet Oncol. (2022) 23:374–81. doi: 10.1016/S1470-2045(22)00062-6 35157829

[14] Koyama T Shimizu T Kojima Y Sudo K Okuma HS Shimoi T. Clinical activity and exploratory resistance mechanism of milademetan, an MDM2 inhibitor, in intimal sarcoma with MDM2 amplification: an open-label phase Ib/II study. Cancer Discov. (2023) 13:1814–25. doi: 10.1158/2159-8290.CD-23-0419 37369013

