# Supplementary figures and images for: Case report: Navigating treatment pathways for cardiac intimal sarcoma with PDGFRβ N666K mutation
Source: Front Oncol. 2024 Apr 5;14:1362347. doi: 10.3389/fonc.2024.1362347 (PMC11026546; doi:10.3389/fonc.2024.1362347)

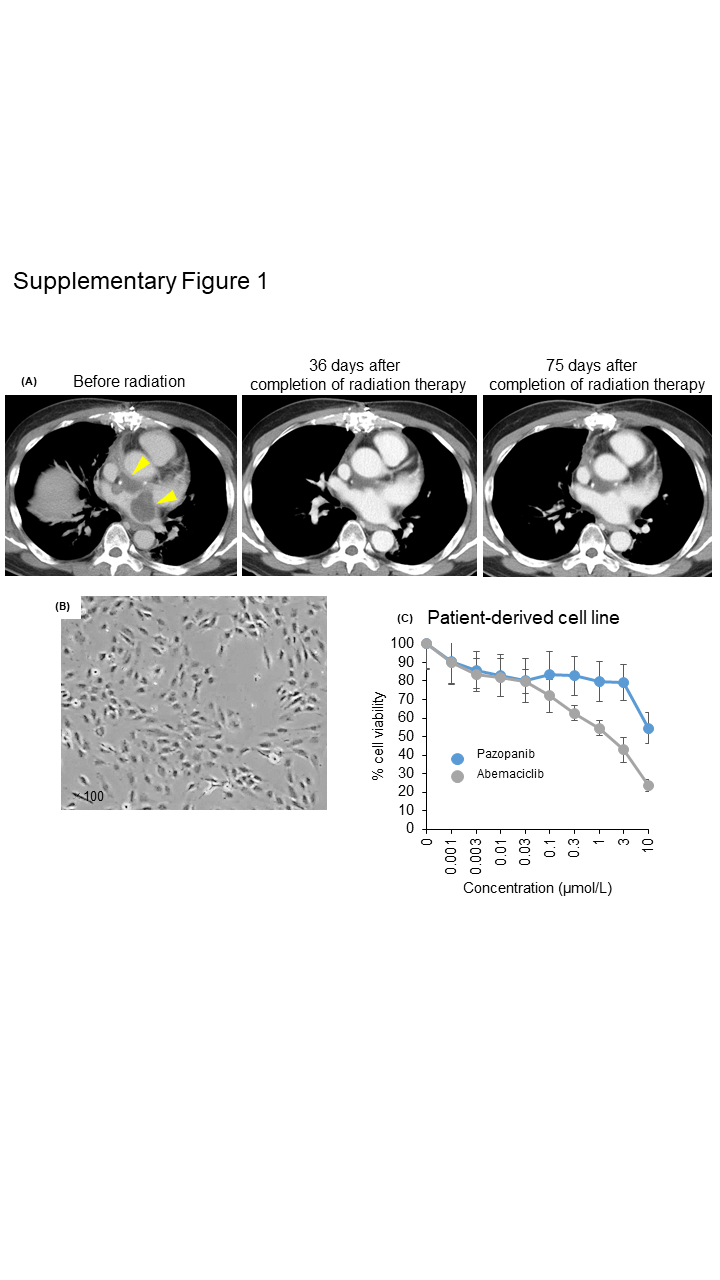

Supplement: Supplementary Figure 1 — The radiographic findings of the local recurrence in the left atrium and the experiment using the recurrence tissue. (A) The yellow arrowheads indicated the recurrence lesions. These lesions responded to palliative radiotherapy. (B) The appearance of the cell line established from the recurrence tissue. (C) The sensitivity of the patient-derived cell line to pazopanib or abemaciclib was determined through cell viability assays. [file Image_1.tif]

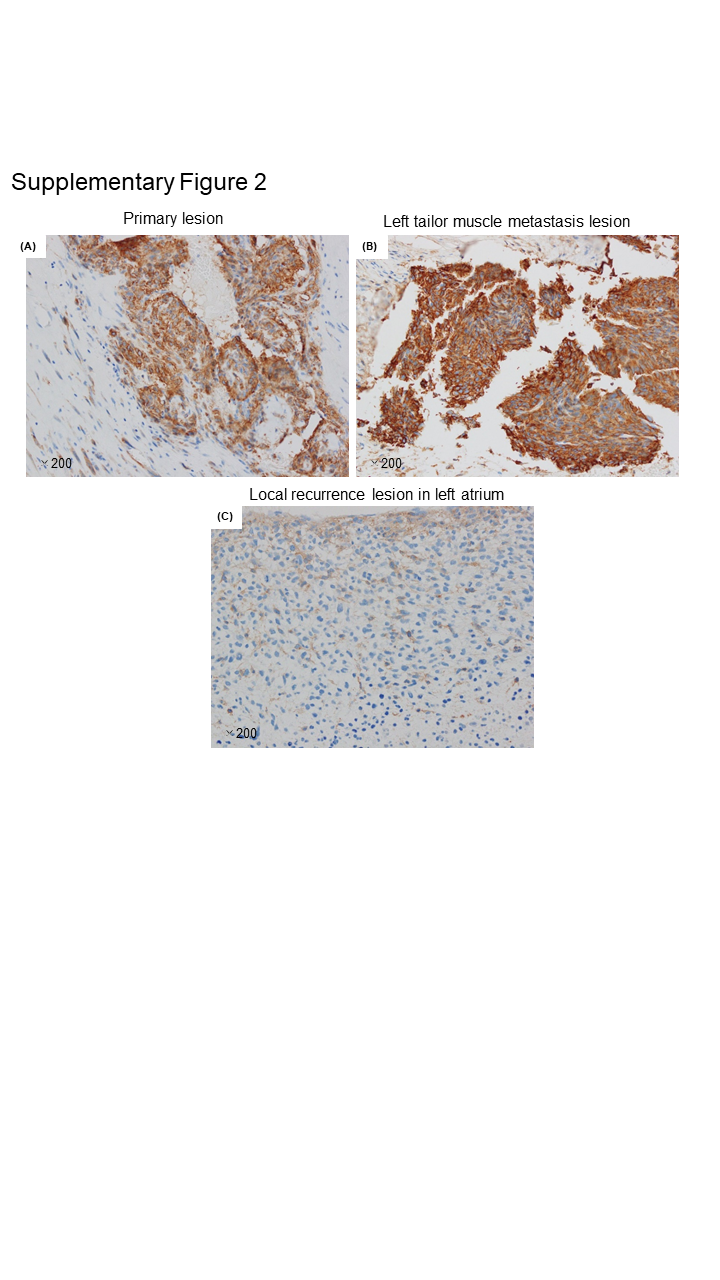

Supplement: Supplementary Figure 2 — Immunohistochemistry staining of PDGFRβ (Cell Signaling Technology; PDGF Receptor β (C82A3) Rabbit mAb #4564). (A) The primary lesion exhibited an adequate expression of PDGFRβ. (B) The left tailor muscle metastasis lesion demonstrated a high expression of PDGFRβ. (C) The local recurrence lesion in the left atrium had a low expression of PDGFRβ. [file Image_2.tif]
